# A Graphene-Based Resistive Pressure Sensor with Record-High Sensitivity in a Wide Pressure Range

**DOI:** 10.1038/srep08603

**Published:** 2015-02-27

**Authors:** He Tian, Yi Shu, Xue-Feng Wang, Mohammad Ali Mohammad, Zhi Bie, Qian-Yi Xie, Cheng Li, Wen-Tian Mi, Yi Yang, Tian-Ling Ren

**Affiliations:** 1 Institute of Microelectronics, Tsinghua University, Beijing 100084, China; 2 Tsinghua National Laboratory for Information Science and Technology (TNList), Tsinghua University, Beijing 100084, China

## Abstract

Pressure sensors are a key component in electronic skin (e-skin) sensing systems. Most reported resistive pressure sensors have a high sensitivity at low pressures (<5 kPa) to enable ultra-sensitive detection. However, the sensitivity drops significantly at high pressures (>5 kPa), which is inadequate for practical applications. For example, actions like a gentle touch and object manipulation have pressures below 10 kPa, and 10–100 kPa, respectively. Maintaining a high sensitivity in a wide pressure range is in great demand. Here, a flexible, wide range and ultra-sensitive resistive pressure sensor with a foam-like structure based on laser-scribed graphene (LSG) is demonstrated. Benefitting from the large spacing between graphene layers and the unique v-shaped microstructure of the LSG, the sensitivity of the pressure sensor is as high as 0.96 kPa^−1^ in a wide pressure range (0 ~ 50 kPa). Considering both sensitivity and pressure sensing range, the pressure sensor developed in this work is the best among all reported pressure sensors to date. A model of the LSG pressure sensor is also established, which agrees well with the experimental results. This work indicates that laser scribed flexible graphene pressure sensors could be widely used for artificial e-skin, medical-sensing, bio-sensing and many other areas.

Flexible e-skin has attracted a great deal of attention due to its ability to sense pressure, potentially initiating vast applications development in health monitoring. Highly sensitive piezoelectric-type nanowire pressure sensors^1^ and capacitive-type micro-structured pressure sensors^2^,^3^ have been reported. However, large scale fabrication of such pressure sensor devices using non-traditional materials presents challenges. Due to its simple device structure and easy fabrication process, resistive-type pressure sensors^4^,^5^,^6^,^7^,^8^,^9^ show tremendous promise for real applications. Most reported resistive pressure sensors^4^,^5^,^6^,^7^,^8^,^9^ have a high sensitivity at low pressures (<5 kPa) to enable ultra-sensitive detection. However, the sensitivity drops significantly at high pressures (>5 kPa), which is inadequate for practical applications. For example, gentle touch and object manipulation generate pressures below 10 kPa and between 10–100 kPa, respectively.

Owing to its single atom layer thickness, graphene was predicted to be a very sensitive platform for pressure sensing^10^,^11^. In fact, graphene based resistive-type pressure sensors have already been developed^12^,^13^,^14^. A single-layer graphene suspended on a cavity^12^ was demonstrated to have a pressure sensing range of up to 100 kPa but with a rather small sensitivity (2.66 × 10^−5^ kPa^−1^). A graphene composite sponge^13^ was reported to have a rather high sensitivity (0.26 kPa^−1^) but it also featured a limited pressure sensing range (<2 kPa). Furthermore, in this device, the sensitivity would drop to 0.03 kPa^−1^ when pressures in excess of 2 kPa were applied. Therefore it is necessary to develop a graphene pressure sensor with a high sensitivity in a wide pressure range. Here a record high sensitivity pressure sensor (0.96 kPa^−1^) in a large range (up to 50 kPa) was developed based on laser-scribed graphene (LSG). The laser-scribed fabrication process enables a large-scale, low cost and time-efficient production of such high performance pressure sensors.

## Results

### Structure and Characterization of the LSG

**Figure 1:**
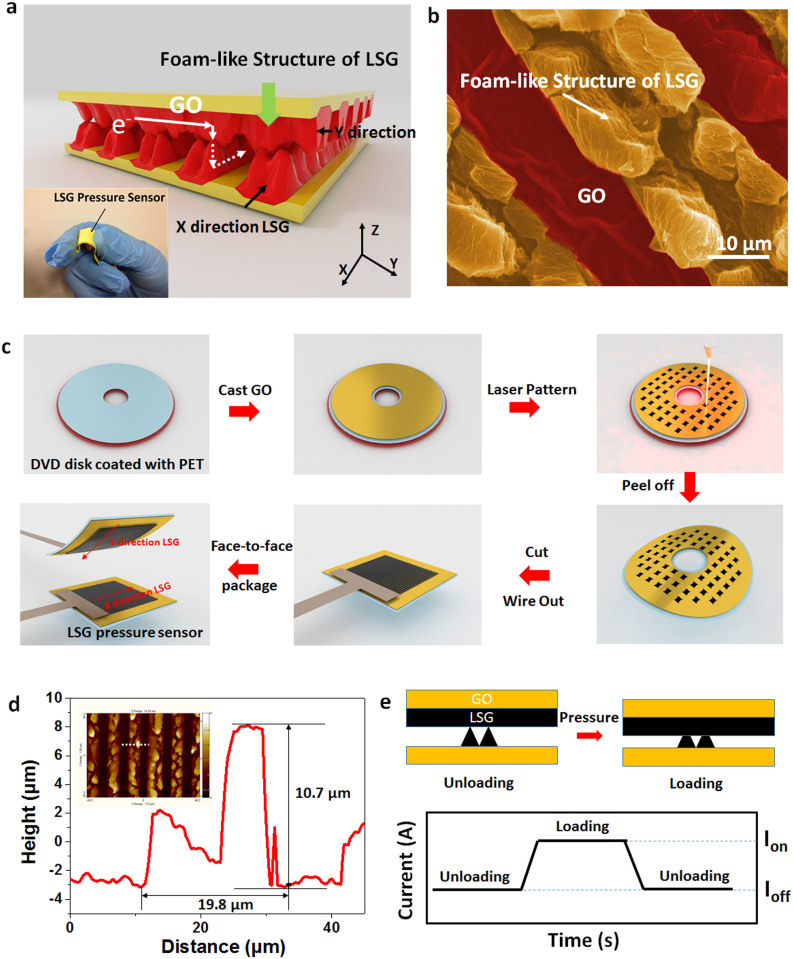
The LSG pressure sensor schematic and microstructure. (a) Cross-bar device structure of the pressure sensor based on the foam-like LSG. Inset showing a flexible LSG pressure sensor in hand. (b) Top view SEM image of the LSG surface in false color. (c) The main fabrication processing steps of the LSG pressure sensor. A DVD burner with a laser-scribing function is used to convert GO into LSG. The upper and lower LSG patterns are perpendicular to each other to form a cross-bar structure. The two pieces of LSG are finally packaged face-to-face. (d) The height profile corresponding to the white line in the inset showing that the height and width of the LSG is 10.7 μm and 19.8 μm, respectively. (e) Schematic illustration of the sensing mechanism and current changes in response to loading and unloading (I_off_: unloading, I_on_: loading).

Figure 1a shows the device structure of the LSG pressure sensor. The pressure sensing core is a crossbar structure consisting of a face-to-face stack of two LSG films (Figure 1b) patterned as v-shaped gratings. Figure 1c shows the fabrication process of the LSG pressure sensor. The LSG film patterning is based on the reduction of graphene oxide (GO) using DVD laser-scribing function^15^,^16^,^17^,^18^. Unlike the dense GO films, the LSG is composed of loosely stacked graphene layers^19^. A 3D profile of the LSG morphology is shown in Figure S1. In Figure 1d, the height profile shows that the height and width of the LSG is 10.7 μm and 19.8 μm with a v-shape.

The LSG pressure sensor is based on resistive change between two pieces of LSG films. The schematic in Fig. 1e shows the device structure and the principle behind current generation. The sensing mechanism can be explained by the force-dependent contact between the two LSG films facing perpendicular to each other. The contact between the two LSG films depend on the applied forces. When applying force on the device, a small compressive deformation can enhance the contact between the two LSG lines and reduce the inter-layer distance of LSG, resulting in more electrical path ways through the crossbar structure. This can cause an increase in current since a fixed voltage bias is applied. After unloading, both LSG films recover to their initial shapes, reducing the contact area and hence the current. The unique microstructure of the LSG is the core feature in this device enabling ultra-sensitive pressure sensing due to a large change in contact area.

### Pressure Response

**Figure 2:**
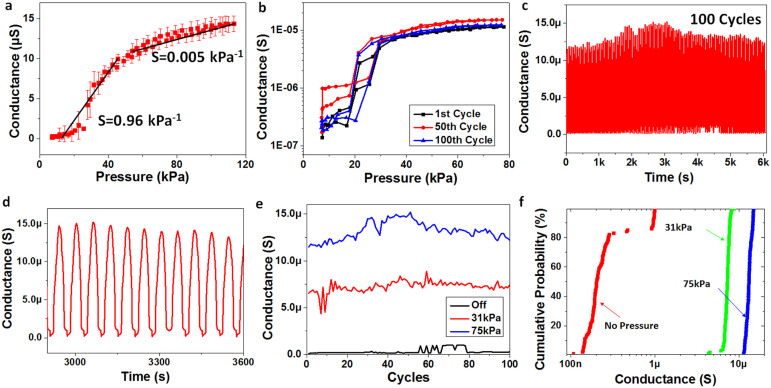
The static pressure response of the LSG pressure sensor. (a) The conductance of the LSG vs. pressure. At low pressures (<50 kPa), the sensitivity is 0.96 kPa^−1^. At high pressures (50 ~ 113 kPa), the sensitivity is 0.005 kPa^−1^. (b) The 1st, 50th and 100th cycles of the pressure response showing repeatability of the performance. (c) The pressure sensor durability test (100 cycles). In each cycle the pressure is swept from 0–75 kPa. (d) A zoomed-in view of the curves in panel (c) after 50 cycles. (e) The pressure sensor conductance over 100 cycles shown for three different pressures. (f) Distribution of the conductance by applied pressure.

In order to test the response of our LSG pressure sensor under static and dynamic forces, a system containing a computer controlled stepping motor, a force sensor and an electrical signal analyzer were used. In this system, static pressure up to 113 kPa and dynamic pressure up to 98 kPa could be loaded. The resistance change could also be simultaneously recorded. As shown in Figure 2a, when a pressure ranging from 0 to 113 kPa is applied, the conductance increases significantly due to enhancement of contact between the two LSG films. The sensitivity can be expressed as:
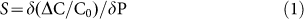

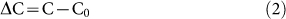
where P is the applied pressure, C is the conductance when pressure is applied on the device, and C_0_ is the conductance under base pressure. It is shown that the sensitivity is as high as 0.96 kPa^−1^ in the low pressure range (<50 kPa) while it lowers to 0.005 kPa^−1^ in the high pressure range. In the low pressure range, there is a significant change of contact-area between the two v-shaped foam-like structures and the density of LSG. After the two v-shaped contacts become stable at high pressure, the area change in contact resistance and density change in LSG are minimal, causing a saturation in sensitivity.

The repeatable performance in the 1^st^, 50^th^ and 100^th^ cycles are shown in Figure 2b. An excellent operational stability of the LSG pressure sensor is demonstrated through a 100 cycle run with a 0 ~ 75 kPa force sweep (Figure 2c). It is noted that a good signal-to-noise ratio (SNR) has been obtained with negligible changes (Figure 2d). Figure 2e shows the testing of 31 kPa pressure, 75 kPa pressure and the off state over 100 cycles. Figure 2f shows the distribution of the conductance over 100 cycles. The 31 kPa and 75 kPa lines are quite uniform, while the off state has a larger fluctuation. This is because a small change in contact area of the two LSG films can induce a large change in conductivity.

**Figure 3:**
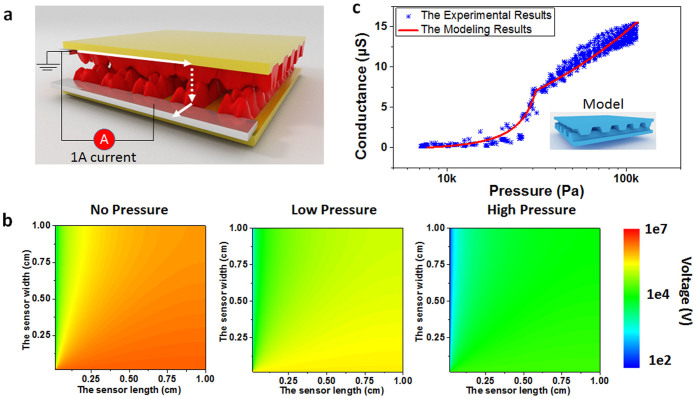
Modeling results of the LSG pressure sensor. (a) The simulation model of the LSG pressure sensor. (b) Voltage drop distribution on the device surface under different pressures. (c) The theoretical and experimental results agree well over 10 cycles.

In order to interpret the relationship between pressure and conductivity, a model is established (see details in supporting information). As shown in Figure 3a, when the two layers touch each other, this can be modelled as a huge net of resistors containing N rows and N columns. The model contains two kinds of resistance (in-plane resistance and inter-plane resistance). In-plane resistance can be referred to as the parallel resistance between two adjacent contact points along the LSG in same plane. The inter-plane resistance can be referred to the resistance perpendicular to the contact point of the resistors. For convenience, it is assumed that the pressure applied on the device is the same everywhere, therefore every inter-plane resistor has the same resistance. For the in-plane resistor, it is assumed that all resistors have the same and constant value *R_p_*. SPICE is used to simulate the relationship between each inter-plane resistance and the total net resistance while C programming language is used to generate the huge simulation file. As shown in Figure S2, the curve where y axis represents the total resistance with in-plane resistance *R_p_*, x axis represents the every inter-plane resistance.

As shown in Figure 3b, the condition where no pressure is applied has the highest voltage drop, which indicates lowest conductivity. With increasing pressure, the voltage drop decreases implying increasing conductivity. As shown in Figure 3c, the experimental results agree well with the theoretical results. The model indicates that the large inter-layer distances of graphene could maintain a high sensitivity in a large pressure range. The saturation may be attributed to the saturation of the material's density.

**Figure 4:**
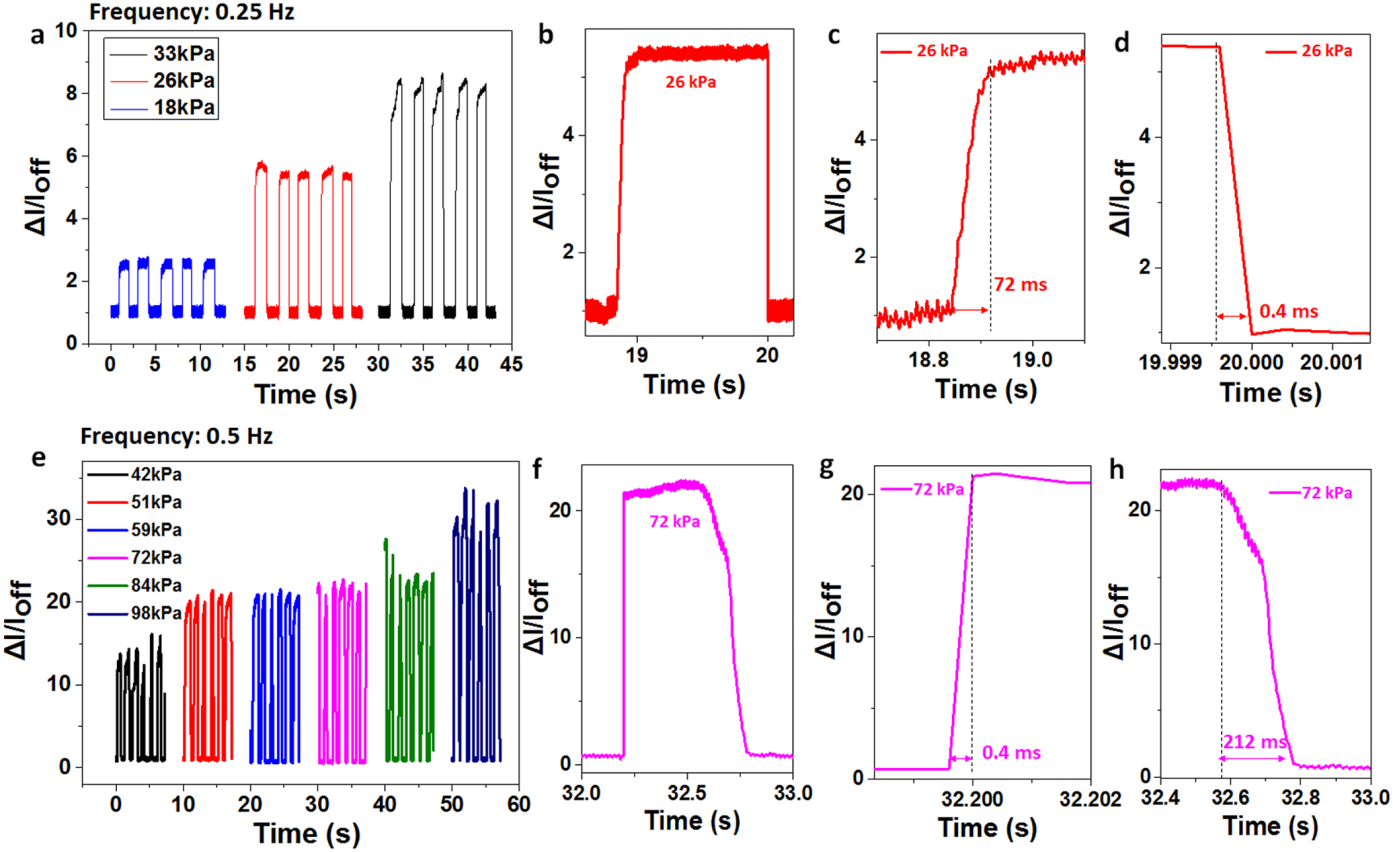
Dynamic pressure response of the LSG pressure sensor. Pressure response at a low frequency (a) for a range of low pressures, (b) showing a single cycle, and the corresponding (c) response time and (d) releasing time. Pressure response at high frequency (e) for a range of high pressures, showing (f) a single cycle, and the corresponding (g) response time and (h) releasing time.

In order to obtain the response time of the LSG pressure sensor, dynamic forces are applied at 0.25 Hz and 0.5 Hz frequencies. In the low pressure range, there is an obvious current change under varying pressure due to its high sensitivity (Figure 4a, 4b). On the other hand, in the high pressure range, there is a smaller difference of the current ratio (Figure 4e, 4f). The typical response time at low and high pressures are 72 ms and 0.4 ms respectively (Fig. 4c, 4g). Meanwhile, the typical releasing time at low and high pressures are 0.4 ms and 212 ms respectively (Fig. 4d, 4h). Note that the 0.4 ms is the minimum resolution of the measurement system. Hence, the response time at high pressure and releasing time at low pressure could be even lower than 0.4 ms. The faster response time at higher pressure and frequency could be explained by the higher force speed, which could result in a more sharp rising edge. Interestingly, the releasing time for a higher pressure and frequency is much longer. This could be explained by the foam structure of LSG. Under a larger force, the LSG is denser, which needs a longer time to recover.

**Figure 5:**
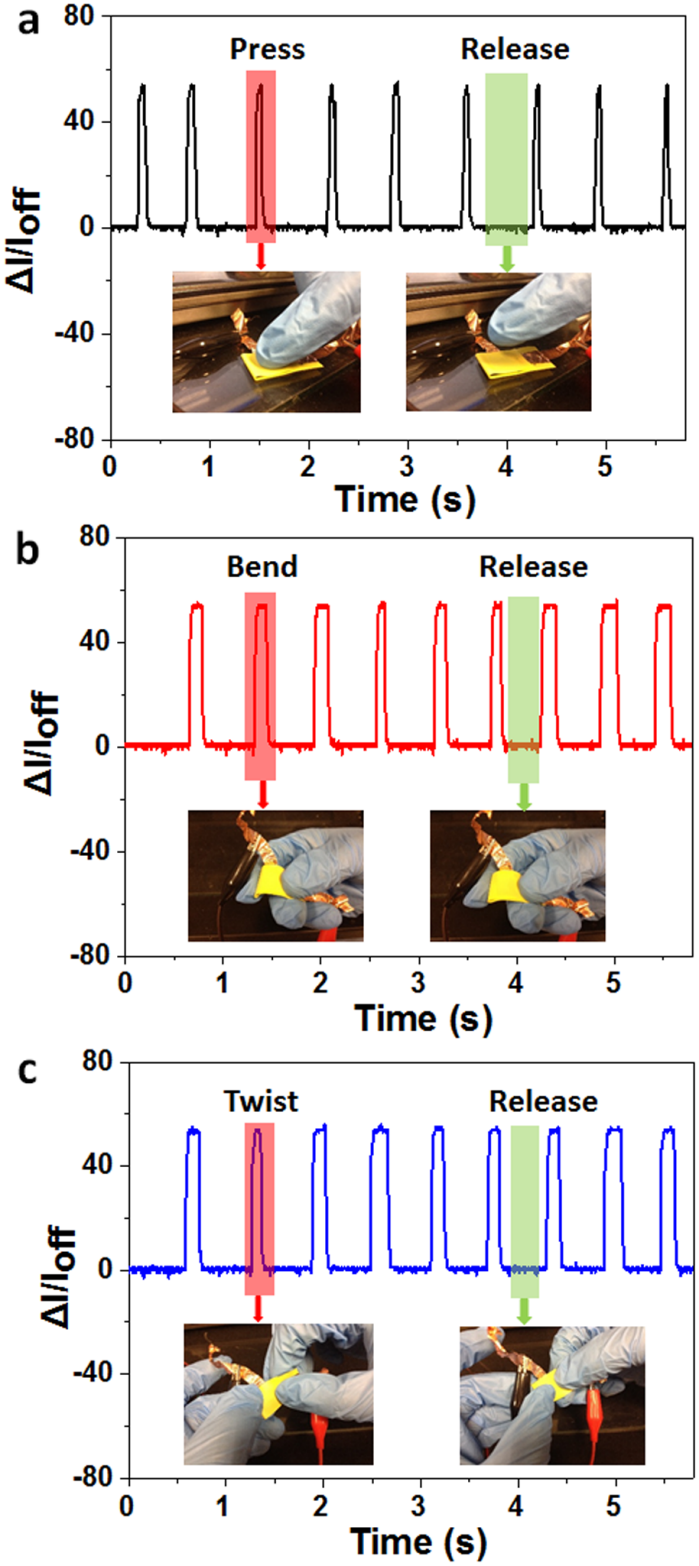
Detection of three types of mechanical forces. Current response plots for dynamic loading and unloading cycles: (a) pressing, (b) bending and (c) twisting. The photos in the inset of each panel demonstrate the actions labelled.

Furthermore, the LSG pressure sensor can also be used to detect pressing (Figure 5a), bending (Figure 5b) and twisting (Figure 5c) forces. High SNRs are obtained in all three types of force measurements, further showing the high sensitivity capability of our LSG pressure sensor.

**Figure 6:**
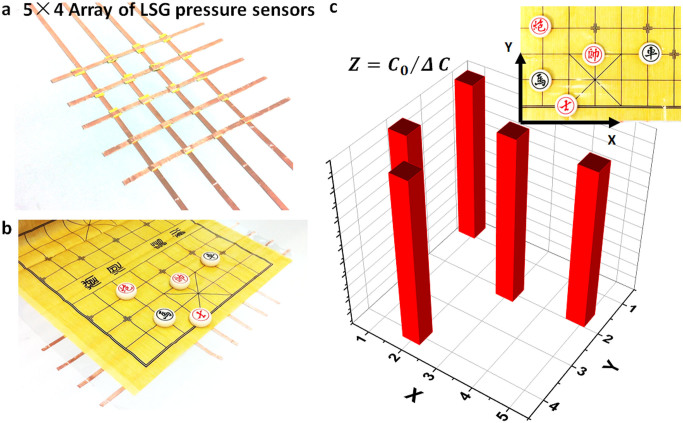
A 5 × 4 array of pressure sensors integrated in a chess board. (a) A 5 × 4 array of the LSG pressure sensors. (b) A Chinese chess board with the pieces placed on the pressure sensor array elements. (c) Electronically identifiable location of chess pieces. The inset shows the actual position of the pieces.

For applications such as human-computer interfaces, arrays of pressure sensors may need to be developed to detect pressure distribution. Based on our LSG pressure sensor, a proof-of-concept pressure array of 20 pixels (5 × 4 elements) is integrated in a Chinese chess board (Figure 6a and 6b). The area of each pixel is 1 cm^2^. The position of the pieces could be identified by testing their conductivity changes as shown in Figure 6c. This data can be fed to a computer for tracking and analysis. The positions obtained from the pressure sensor array agree with the positions shown in the inset of Figure 6c.

**Figure 7:**
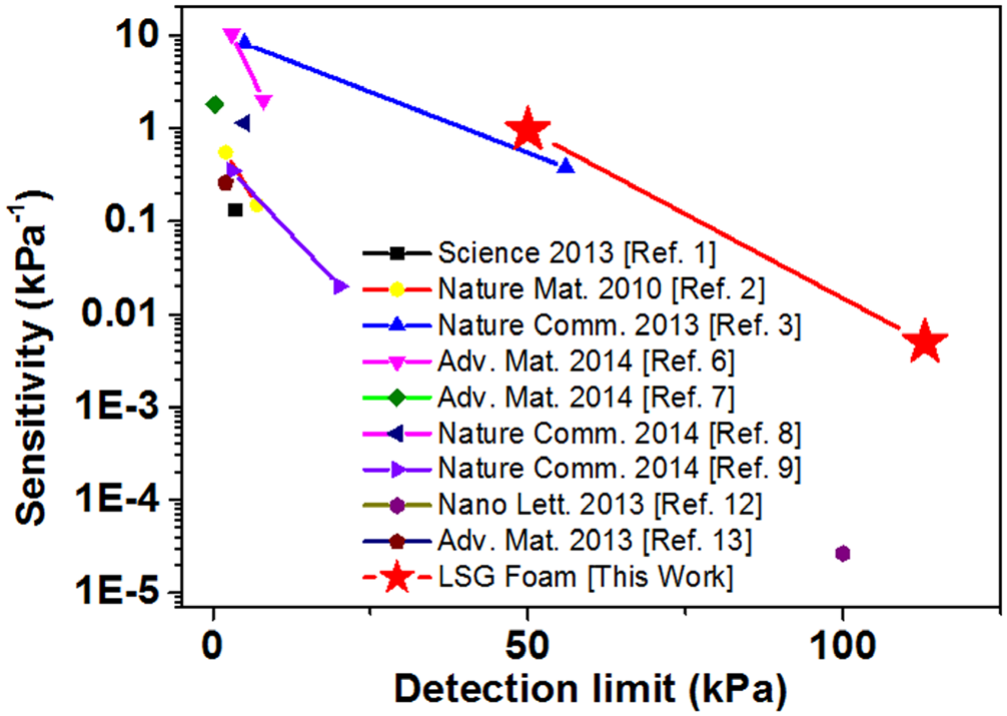
Comparison of the LSG pressure sensor to other reported pressure sensors. As compared to all reported nano-structured pressure sensors, our LSG pressure sensor performs the best when simultaneously considering both sensitivity and sensing range^1^,^2^,^3^,^6^,^7^,^8^,^9^,^12^,^13^.

There are three main types of pressure sensors: piezoelectric, capacitive, and resistive. The sensitivity and pressure sensing range are the two key parameters used to evaluate the performance of the pressure sensor. All representative types of high performance pressure sensors are plotted together with the pressure limit as the horizontal axis and sensitivity as the vertical axis. In such a way, the point in upper right corner shows excellent sensitivity and pressure sensing range simultaneously. As shown in Figure 7, the sensitivity combined with the sensing range of our LSG pressure sensor is the best among all reported pressure sensors to date^1^,^2^,^3^,^6^,^7^,^8^,^9^,^12^,^13^.

## Conclusion

A flexible, ultra-sensitive resistive pressure sensor based on LSG is demonstrated. The sensitivity of the pressure sensor is as high as 0.96 kPa^−1^ in a wide pressure range (0 ~ 50 kPa). Gaining from the large inter-spacing between graphene layers and the unique v-shaped microstructure of the LSG, the sensitivity combined with the pressure sensing range is the best among all reported pressure sensors to-date. Moreover, dynamic pressure testing shows that the response time could be as fast as 0.4 ms under high pressure. The theoretical model formulated in this work agrees well with the experimental results and explains the different regimes of operation. Laser scribing technology enables rapid, large-scale, and low-cost production of the LSG which is a major advantage in the development of pressure sensors from a commercialization point-of-view. This work indicates that the LSG pressure sensors hold great promise for e-skin and other sensing applications.

## Methods

### Synthesis

A 2 mg/ml Graphene Oxide (GO) dispersion in water was purchased from XFNANO Materials Tech CO., Ltd (Nanjing, China). Graphite powder was used to synthesize GO solutions using a common Hummers method^20^. About 10 mL GO solution was drop-casted on the surface of a LightScribe DVD disc. The GO solution was left overnight to dry on the DVD disc. After that, the GO coated DVD disc was patterned by a light-scribe DVD Drive (HP Inc. 557S) inducing a local reduction of the GO into LSG. The designs were made using the Nero Start Smart software.

### Characterization

The surface morphology of the LSG pressure sensor is observed using a Quanta FEG 450 SEM (FEI Inc.). The 2D image of the LSG surface is captured by a white light interference microscope microXAM-1200 (MapVue AE Inc.).

### LSG pressure sensor fabrication

After fabrication of the LSG film, two pieces of the LSG film are cut with 1 × 1 cm^2^ dimensions. These pieces are assembled face-to-face with a small gap. Note that the orientation of these two pieces should be perpendicular to each other. Finally, each of the LSG films are wired out using copper wires and packaged with tape.

## Author Contributions

H.T. conceived the project, did the experiment and wrote the paper. Y.S. assisted in the experiments. X.-F.W. performed the simulation. M.A.M., W.-T.M., C.L., Y.Y., Z.B. and Q.-Y.X. assisted in the experiments, analysis, and revised the manuscript. T.-L.R. supervised the project.

## Supplementary Material

Supplementary Information Supporting Information
