# Supplementary material for: A Graphene-Based Resistive Pressure Sensor with Record-High Sensitivity in a Wide Pressure Range
Source: Sci Rep. 2015 Feb 27;5:8603. doi: 10.1038/srep08603 (PMC4342573; doi:10.1038/srep08603)
Supplement: Supplementary Information — Supporting Information [file srep08603-s1.pdf]

A Graphene-Based Resistive Pressure Sensor with Record-High Sensitivity in a Wide Pressure Range

--Supplementary Information

He Tian,[1,2,†] Yi Shu,[1,2,†] Xue-Feng Wang,[1,2,†], Mohammad Ali Mohammad,[1,2,†]

Zhi Bie,[1,2] Qian-Yi Xie,[1,2] Cheng Li,[1,2] Wen-Tian Mi,[1,2] Yi Yang,[1,2] Tian-Ling Ren,[1,2,*]

[1]Institute of Microelectronics, Tsinghua University, Beijing 100084, China

[2]Tsinghua National Laboratory for Information Science and Technology (TNList), Tsinghua University, Beijing 100084, China

[†]These authors contributed equally to this work

*Corresponding Author E-mail:

RenTL@tsinghua.edu.cn

I. LSG microstructure

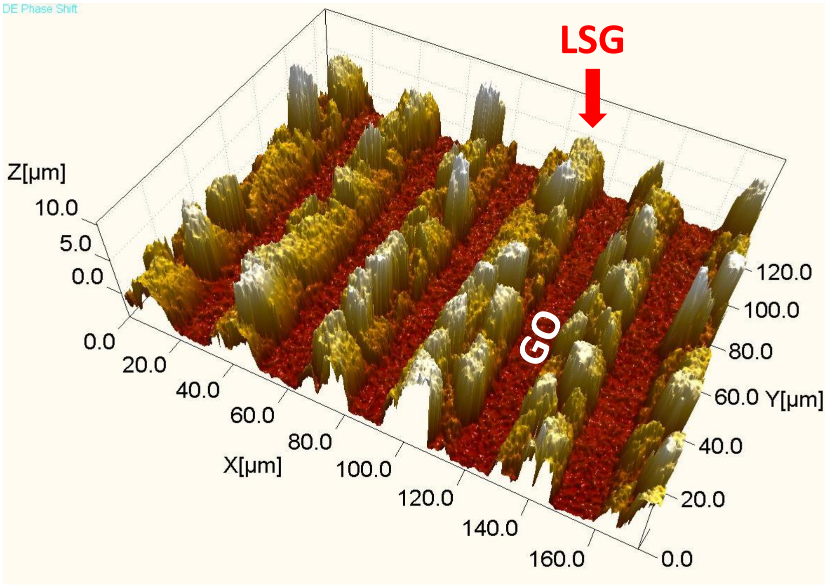

Figure S1. A 3D profile of the LSG morphology captured by a white light interference microscope.

II. The model of the LSG pressure sensor

Here we discuss the model formulated to interpret the relationship between pressure and conductivity in the LSG pressure sensor.

According to Figure 1a, the contact between two LSG layers can be modelled as a huge net of resistors containing N rows and N columns. The modelling contains two kinds of resistance (in-plane resistance and inter-plane resistance). The in-plane resistance can be referred to as the parallel resistance between two adjacent contacting points along the LSG in the same plane. The inter-plane resistance can be referred to the resistance perpendicular to the contact point of the resistors. For convenience, it is assumed that the pressure applied everywhere on the device is the same, therefore every inter-plane resistor has the same resistance. For the in-plane resistance, it is assumed that all have the same and unchangeable value R_p.

SPICE is used to simulate the relationship between every inter-plane resistance and the total net resistance while C programming language is used to generate the huge simulation file. In Figure S2, the vertical axis represents the total in-plane resistance,

while the horizontal axis represents the each inter-plane resistance.

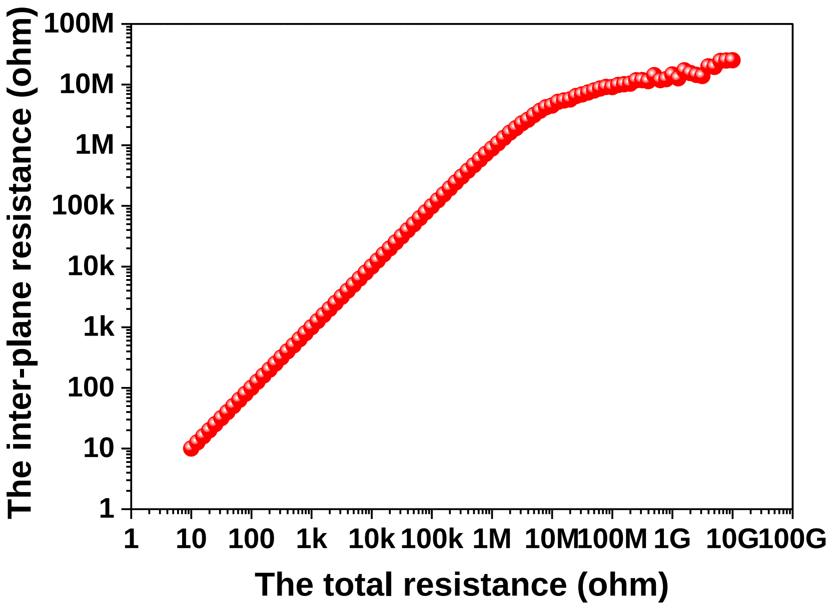

Figure S2. Theoretical results of the relationship between the inter-plane resistance and the total resistance

The relationship between the inter-plane resistance and the force applied on the device can be calculated as per the analysis shown below:

1. Pressure and displacement formula calculation：

The model is illustrated in Figure S3.

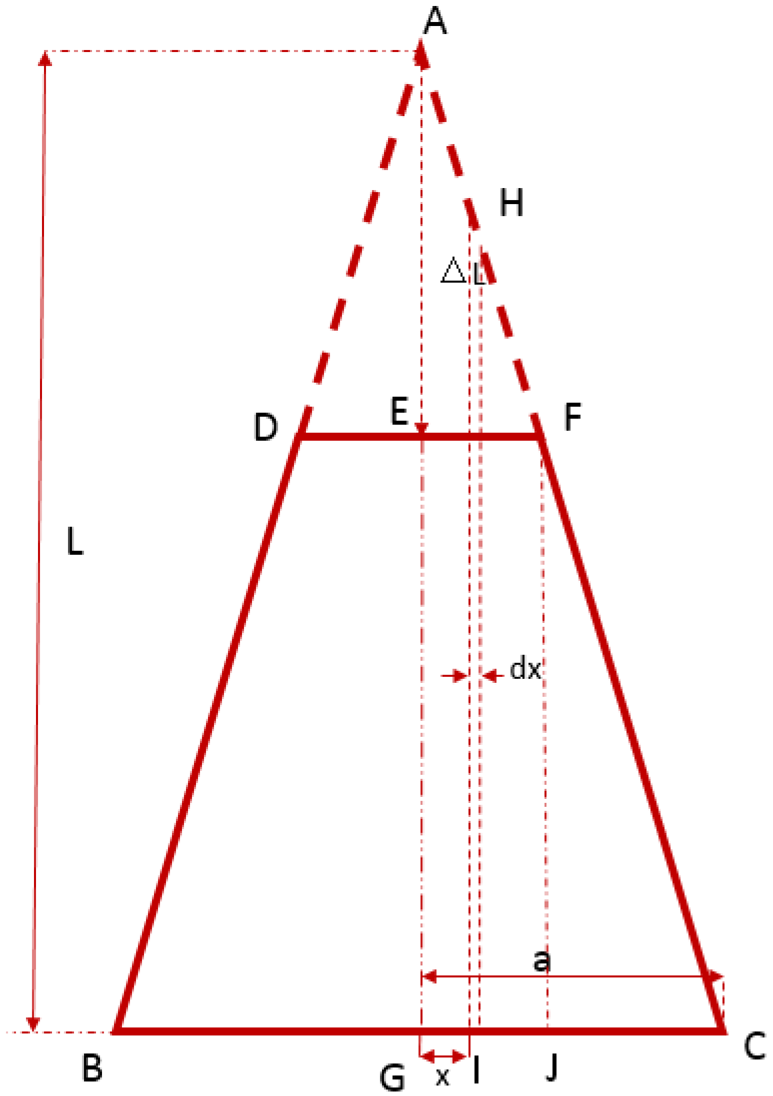

Figure S3. Geometrical modelling of the inter-plane contact

Consider the diagram in Fig. S3 as a cross-section of a single LSG trace. One-half of the base length (unit length) is labelled a, the total height before applying force is L, and the width of a given side is b (not displayed in the diagram). From the micro-structure, we can model this foam-like structure as an open-door foam, which satisfies the following formula:[S1]

$$\frac{E^*}{E} = \left(\frac{\rho^*}{\rho}\right)^2 \qquad (1)$$

Where E and E^* are the elastic modulus before and after applying pressure. The

variables ρ^* and ρ are the density of the foam hole. In order to simplify the model, we can use eq. (1) to obtain the dx as shown in Figure S3. Therefore we can get

$$\frac{E^*}{E} = \left(\frac{\rho^*}{\rho}\right)^2 \approx \left(\frac{V_{HI}}{V_{KI}}\right)^2 \approx \left(\frac{HI}{KI}\right)^2 = \left(\frac{a-x}{a}\frac{L}{L-\Delta L}\right)^2 \qquad (2)$$

According to Young`s theorem

$$\frac{F}{S} = E\frac{\Delta l}{l} \qquad (3)$$

where F is the force, S is the cross-sectional area, l is the total column length, and Δl is the scratch length. In order to analyze the target structure, we first analyze the dx located in x. Therefore, $F = dF$, $S = b\frac{\Delta L}{L}dx$, $l = \frac{a-x}{a}L$, and $\Delta l = \Delta L - \frac{x}{a}L$.

Combine these relations with equation (2), we obtain:

$$\frac{dF}{dx} = bE\left(\frac{a-x}{a}\right)\frac{L}{(L-\Delta L)^2}\left(\Delta L - \frac{x}{a}L\right)dx \qquad (4)$$

Integrating the above equation yields an F vs Δl relation.

$$F = 2\int_0^{a\Delta L/L} bE\left(\frac{a-x}{a}\right)\frac{L}{(L-\Delta L)^2}\left(\Delta L - \frac{x}{a}L\right) \qquad (5)$$

$$F = \frac{Eab\Delta L^2(3L-\Delta L)}{3L(L-\Delta L)^2} \qquad (6)$$

When ΔL is very small, $L - \Delta L \approx L$, and $3L - \Delta L \approx 3L$. Using these approximations, eq. (6) can be simplified as follows:

$$F = \frac{Eab\Delta l^2}{L^2} \qquad (7)$$

2. Inter-plane resistance vs. force

In order to obtain the inter-plane resistance vs. force relationship, we must first obtain the displacement vs. resistance relation. The basic formula in eq. (8) can be used for this purpose:

$$\mathrm{R} = \rho \frac{L}{S} \tag{8}$$

According to our model, we can rewrite eq. (8) as follows

$$dR = \rho \frac{dx}{\frac{x}{l}ab}$$

$$R = \int_{\Delta L}^{L} \rho \frac{dx}{\frac{x}{L}ab\frac{\Delta L}{L}}$$

$$R = \frac{\rho L^2}{ab\Delta L} ln \frac{L}{\Delta L} \tag{9}$$

Therefore the relationship between R and ΔL has been obtained. Further, we combine eq. (9) and (6) to get the relationship between R and F.

3. According to ref. [S2], the electrical resistivity of the foam-like LSG obeys the following relationship:

$$\frac{\rho}{\rho_0} = \frac{2K\ (1-\theta}{2K+\theta} \tag{10}$$

Where ρ / ρ_0 is the relative electrical resistivity, θ is the porosity, and K is the coefficient related to the geometric structure and other properties of foam-like materials.

When we apply force on the material, the air is squeezed-out of the pores. The volume and porosity prior to applying force is given by V_t and θ_0, respectively. The volume and displacement after applying force is given by V and ΔL, respectively. The formula can be re-written as follows:

$$\theta = \frac{\theta_0 V_t - V}{V_t - V} = \frac{\theta_0 - \frac{V}{V_t}}{1 - \frac{V}{V_t}} \tag{11}$$

According to a geometrical relationship, $\frac{V}{V_t}$ can be rewritten as:

$$\frac{V}{V_t} = \left(\frac{\Delta L}{L}\right)^2 \tag{12}$$

Combining eq. (11) with eq. (12), we obtain:

$$\theta = \frac{\theta_0 L^2 - \Delta L^2}{L^2 - \Delta L^2} \tag{13}$$

When a force is applied to the material, the structure and some properties of the material change. So K is the variable during the processing. The following model is supposed:

$$K = K_0 + \frac{\alpha L}{L - \Delta L} \tag{14}$$

Where K_0 and a is constant.

However if the ΔL is relatively large, then ρ becomes ρ_t (a constant) i.e., the electrical resistivity doesn't change if the material is condensed enough.

Fig. S4 presents the actual experimental results and the curve generated through modelling.

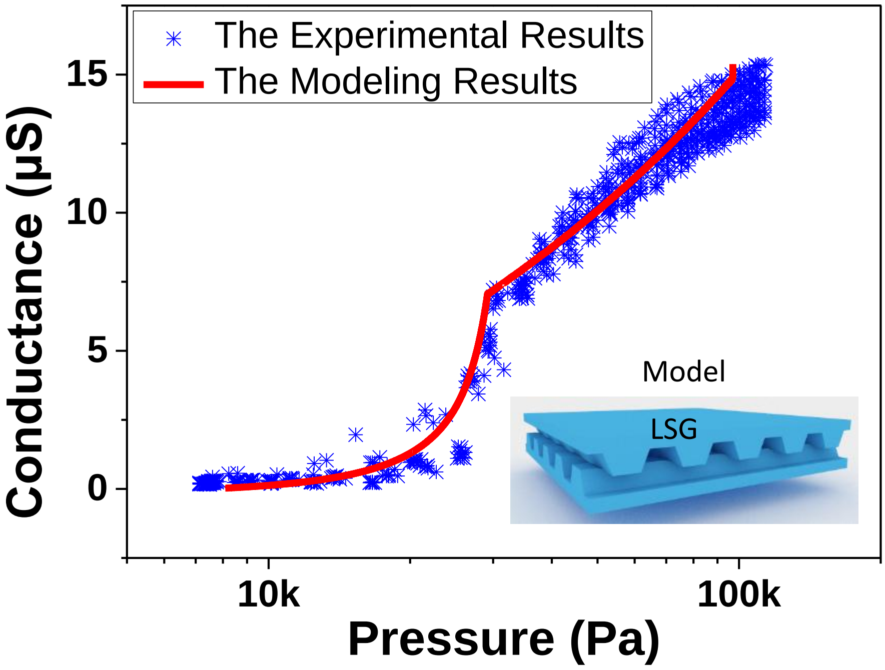

Figure S4. Comparing experimental and theoretical data regarding the

relationship between the applied force and total conductivity

SUPPLEMENTARY TABLES

Table I. Simulation values

Symbol	Unit	Description	value
N		The number of rows and columns used in modeling	64
R_p	MΩ	In-plane resistance	37.0
ρ_0	Ω/m	Initial resistivity without pressure	187
E	Pa	Young's modulus of material	4.25×10^{6}
a,b	m	The length of the LSG	2×10^{-5}
L	m	The height of the LSG	1×10^{-5}
θ_0		The initial porosity	0.8
K_0		The constant in eq. (14)	0.15
α		The constant in eq. (14)	1.3×10^{-6}
ρ_t	Ω/m	The final unchanged resistivity	104

Table II. Comparison of the LSG pressure sensor with other reported pressure sensors.

Types of devices	Structure	Material	Sensitivity (kPa^{-1})	Detection limit (kPa)	References
Piezoelectric	Nanowire	ZnO	0.131	3.5	Science 2013 [1]
Capacitive	Pyramid	PDMS	0.55<br>0.15	2<br>7	Nature Materials 2010 [2]
Capacitive	Patterned	PDMS	8.2<br>0.38	5<br>56	Nature communications 2013, 4, 1859. [3]
Resistive	Pyramid	PDMS	10.32<br>2.02	3<br>8	Advanced materials 2014, 1. [6]
Resistive	Patterned	PDMS	1.8	0.3	Advanced materials 2014, 26, 1336.[7]
Resistive	Nanowire	Au	1.14	5	Nature Communications 2014, 5, 1 [8]
Resistive	Hollow-sphere	conducting polymer thin film	0.35<br>0.02	3<br>20	Nature Communications 2014, 5, 3002. [9]
Resistive	Suspended	CVD Graphene	2.66×10^{-5}	100	Nano Letters 2013 [12]
Resistive	Sponge	Graphene–Polyurethane	0.26	2	Advanced Materials 2013 [13]
Resistive	Foam	Laser-Scribed Graphene	0.96<br>0.005	50<br>113	This Work

REFERENCES

S1. Huang P Y.Principles of Powder Metallurgy［M］，Metallurgical Industry Press，Beijing，1997.

S2. Gibson L J, Ashby M F. Cellular solids: structure and properties[M]. Cambridge University Press, 1999.
